# Effectiveness of a Single Chair Side Application of NovaMin^®^ [Calcium Sodium Phosphosilicate] in the Treatment of Dentine Hypersensitivity following Ultrasonic Scaling—A Randomized Controlled Trial

**DOI:** 10.3390/ma16041329

**Published:** 2023-02-04

**Authors:** Jeeth Janardhan Rai, Saurabh Chaturvedi, Shankar T. Gokhale, Raghavendra Reddy Nagate, Saad M. Al-Qahtani, Mohammad Al. Magbol, Shashit Shetty Bavabeedu, Mohamed Fadul A. Elagib, Vatsala Venkataram, Mudita Chaturvedi

**Affiliations:** 1 Department of Periodontology, Bharati Vidyapeeth Dental College and Hospital, Sangli 416406, Maharastra, India; 2 Department of Prosthetic Dentistry, College of Dentistry, King Khalid University, Abha 61421, Saudi Arabia; 3 Department of Periodontics and Community Dental Sciences, College of Dentistry, King Khalid University, Abha 61421, Saudi Arabia; 4 Restorative Dental Sciences, College of Dentistry, King Khalid University, Abha 61421, Saudi Arabia; 5 Department of Pedodontics & Preventive Dentistry, KVG Dental College & Hospital, Sullia 574327, Karnataka, India; 6 Independent Researcher, Bhopal 462008, Madhya Pradesh, India

**Keywords:** NovaMin^®^, bioactive glass, dentinal hypersensitivity, calcium sodium phosphosilicate

## Abstract

Dentinal hypersensitivity or cervical dentinal sensitivity is one of the commonest clinical problems. The aim of this randomized controlled trial was to evaluate the effectiveness of a single chair side application of 100% pure calcium sodium phosphosilicate (NovaMin^®^) in reducing dentin hypersensitivity following ultrasonic scaling as evaluated on a visual analogue scale (VAS). The study included 50 subjects who were selected based on an evaluation of dentinal hypersensitivity on a VAS carried out using a metered air blast from a three-way syringe and divided into two groups (*n* = 25/group); i.e., the test group (Group A) received the NovaMin^®^ paste and the control group (Group B) received a placebo paste made from pumice. All the 50 subjects included in the study were had VAS scores of 3 or more. The NovaMin^®^ powder mixed with distilled water was applied. Dentinal hypersensitivity was reassessed immediately and after 1, 2 and 4 weeks after the procedure. Results showed that the percentage reduction of dentinal hypersensitivity following a single application of NovaMin^®^ in powder form was about 76.38% immediately, 67.72% one week postoperatively, 52.76% two weeks postoperatively and 26.78% four weeks postoperatively. It can be concluded from the results of the current clinical study demonstrated that a single chair side application of NovaMin^®^ in powder form has a significant and immediate reduction in dentinal hypersensitivity, which lasted nearly for four weeks.

## 1. Introduction

The hypersensitivity arising from the tooth as a whole or especially from the cervical part of the tooth is one of the commonest clinical problems. It is characterized by acute, non-spontaneous, short or long-lasting pain arising from exposed dentine in response to stimuli typically thermal, evaporative, tactile, osmotic or chemical, and which cannot be corelated to pathology arising from a dental defect of any other origin form [1,2,3]. Hypersensitivity is attributed to the general increase in exposed dentine over the root surfaces of the teeth resulting from periodontal disease, toothbrush abrasion or a pointed load of repetitive stress at the thin enamel near the cementoenamel junction [4,5].

The age for occurrence of dentinal hypersensitivity generally ranges from early teenagers up to 70-year-old individuals [6]. However, the increased incidence is generally reported to be between the range of 20 to 40 years old, and it is more prevalent in females [6,7,8]. The buccal cervical area has the highest predilection for dentinal hypersensitivity [7]. Several studies have shown that the prevalence rate of dentinal hypersensitivity varies from 2 to 85% [9,10]. Dentinal hypersensitivity has also been shown to have a negative effect on psychological and emotional wellbeing. The causes of dentine hypersensitivity are basically from three factors, viz.: (i) recession leading to root exposure, (ii) formation of porosities at the surface followed by exposure of nascent dentinal tubules and (iii) susceptibility of pulp nerves to alteration of movement of the fluid in the dentine. Overall, it appears that it is the combination of more than one of the above-mentioned factors that might lead to dentinal hypersensitivity, rather than just the one factor responsible for it. Irrespective of etiologies for exposure to dentine, the apparent cause and effect is the open dentinal tubules which provide a direct link between the oral environment and the pulp chamber. The occurrence of hypersensitivity will be very unlikely when the dentinal tubules are fully covered by overlying enamel and cementum. The apertures of the dentinal tubules around the sensitive teeth are patent, and this causes the availability of stimuli close to the nerves. The hydrodynamic theory put forth by Brannstrom and colleagues [11,12] is by far the most widely accepted explanation for dentinal hypersensitivity. According to this theory’s postulates, temperature, physical, or osmotic changes can upset the fluids in the dentinal tubules, and these fluid movements can activate a baroreceptor and cause a neural discharge.

Periodontal therapy including surgical and non-surgical therapies are routinely carried out by dentists. Patients often report an immediate increase in dentinal sensitivity during the procedure or post-procedure once the anesthetic effect wears off. Studies have shown that routine periodontal therapies like ultrasonic scaling have been shown to cause an increase in dentinal sensitivity [13,14,15,16,17]. The duration required for the dentinal sensitivity to slowly subside would be around two weeks [14]. Sensitivity following ultrasonic scaling may be attributed to the inadvertent damage occurring to the tooth structure exposing the dentine to the oral cavity. It is also linked to the presence of calculus which occludes the tubules and can lead to the exposure of dentinal tubules following its removal from that area.

There are various treatment modalities presently in vogue towards managing and preventing dentinal hypersensitivity, including nerve desensitization, anti-inflammatory agents, plugging dentinal tubules, dentinal sealers, periodontal soft tissue grafting, crown placement/restorative materials and LASERS (light amplification by the stimulated emission of radiation) [18,19].

Out of all of these methods, the one most frequently used to treat dentinal hypersensitivity is plugging the exposed dentinal tubules with a substance that forms a deposition layer and mechanically occludes dentinal tubules. This method lessens sensitivity by putting a barrier between the flow of pulpal fluid and dentine. The different substances used in this route include potassium oxalate, sodium monofluorophosphate, calcium hydroxide, ferrous oxide, potassium oxalate, sodium monofluorophosphate, sodium fluoride, sodium fluoride/stannous fluoride combination, stannous fluoride, strontium chloride, formaldehyde, glutaraldehyde, silver nitrate, strontium chloride, hexahydrate, casein phosphopeptides, burnishing fluoride and iontophoresis [8,20,21,22,23,24,25,26,27].

Numerous studies have been conducted on the use of NovaMin^®^, a type of bioactive glass that contains calcium sodium phosphosilicate, to alleviate dentinal sensitivity [28,29,30,31,32,33,34,35,36,37]. The brand name NovaMin^®^ refers to a particulate bioactive glass that was created and patented by NovaMin^®^ Technology, Inc. and is used in dental care products to remineralize teeth (Alachua, FL, USA). NovaMin^®^ is made up of 45% SiO_2_, 24.5% Na_2_O, 24.5% CaO, and 6% P_2_O_5_ in aqueous solutions. Calcium, phosphorus, silica, and sodium, which are essential for the mineralization of bones and teeth, are provided by NovaMin^®^ in an ionic form. When these substances are exposed to bodily fluids, they react strongly and deposit hydroxyapatite, a mineral that is chemically similar to the minerals found in bone, enamel, and dentin.

When NovaMin^®^ is exposed to an aqueous environment, the physical occlusion of NovaMin^®^ particles begins. The particles’ sodium ions start exchanging with hydrogen cations rapidly. This quick release of ions enables the release of phosphate and calcium ions from the structure of the NovaMin^®^ particle. As soon as the particles are exposed, a number of early reactions start to take place. As long as the particles are exposed to an aqueous environment, the calcium and phosphate ions continue to be released. When NovaMin^®^ is first exposed, the release of sodium causes a localized, brief rise in pH. By precipitating the calcium and phosphate ions from the NovaMin^®^ particle, as well as the calcium and phosphorus present in saliva, this rise in pH aids in the formation of a calcium phosphate layer. The layer crystallizes into hydroxycarbonate apatite, which is chemically and structurally identical to biological apatite, as the particle reactions and calcium and phosphorus complex deposition proceed. The physical blockage of dentinal tubules caused by the interaction of the remaining NovaMin^®^ particles and the hydroxycarbonate apatite layer will reduce hypersensitivity [28,29].

Clinical trials have shown the efficacy of NovaMin^®^-containing toothpaste in significantly reducing dentinal hypersensitivity over an eight-week period with follow-up interviews up to twelve weeks after cessation of product use [33]. Another study comparing NovaMin^®^-containing toothpaste with other desensitizing toothpastes showed that NovaMin^®^-containing toothpaste performs as well as or better than the positive control with respect to rapidly relieving tooth hypersensitivity after two weeks and six weeks of daily use [34]. There are a few studies showing the efficacy of the NovaMin^®^ powder applied after ultrasonic scaling followed by using the NovaMin^®^ toothpaste at home later [36,37]. A literature search shows no study mentioning the effects of a single use of NovaMin^®^ following ultrasonic scaling on dentinal sensitivity.

The aim of the current randomized clinical trial was to evaluate the efficacy of single chair-side application of NovaMin^®^ in powder form to reduce dentin hypersensitivity as evaluated on a visual analogue scale (VAS) [38] over a four-week period.

## 2. Materials and Methods

### 2.1. Study Design

A double-blind parallel arm randomized controlled study was planned to evaluate the effectiveness of single chair-side application of NovaMin^®^ in powder form and to compare with pumice as a negative control to reduce dentin hypersensitivity as evaluated on a visual analogue scale (VAS) following ultrasonic scaling. The study was conducted in accordance with the Declaration of Helsinki, and the protocol was approved by the Ethics Committee of the Institute, College of Dentistry, King Khalid University, Abha, KSA. (IRB/KKUCOD/ETH/2019-20/012). Before taking part in the study, all subjects provided their informed consent for inclusion and the study protocol was created.

### 2.2. Study Subjects and Sample Size

Sample size (*n*) was calculated by using formula

$$\;n=\frac{2{\left(\mathrm{Standard}\;\mathrm{deviation}\right)}^{2}}{{\left(\mathrm{Effect}\;\mathrm{size}\right)}^{2}}{\left(Z_{\alpha /2}+Z_{1-\beta }\right)}^{2}$$

where *Z*_α/2_ = 1.96 and *Z*_1−*β*_ = 0.842, respectively, represent the 95% confidence intervals based on the usual normal distribution with an 80% study power. To identify a significant difference in dentin hypersensitivity following intervention with NovaMin^®^, with an effect size of 0.50 and a standard deviation of 0.45 from the pilot trial utilizing ten patients, at least seventeen subjects were required. To reach the final sample size, 10% of *n* is added to account for dropouts. As a result, we kept *n* = 25 patients in each group even though the minimal sample size needed for each group was *n* = 20.

The study was conducted on 50 subjects (aged between 25 to 40 years) having mild to moderate localized dentinal hypersensitivity in the Department of Periodontology. The patients were divided randomly into two groups i.e., the test group (Group A) receiving NovaMin^®^ paste and the control group (Group B) receiving a placebo paste made from pumice. Dentin hypersensitivity was measured in each selected subject. Each group was subdivided again based on the time from T0 to T5. T0 was before the start of the treatment, T1 was immediately after ultrasonic scaling, T2 was post-treatment, T3 was one week after treatment, T4 was 2 weeks after treatment and T5 was 4 weeks after treatment.

### 2.3. Preparation of NovaMin^®^ Paste and Pumice Paste

**Figure 1:**
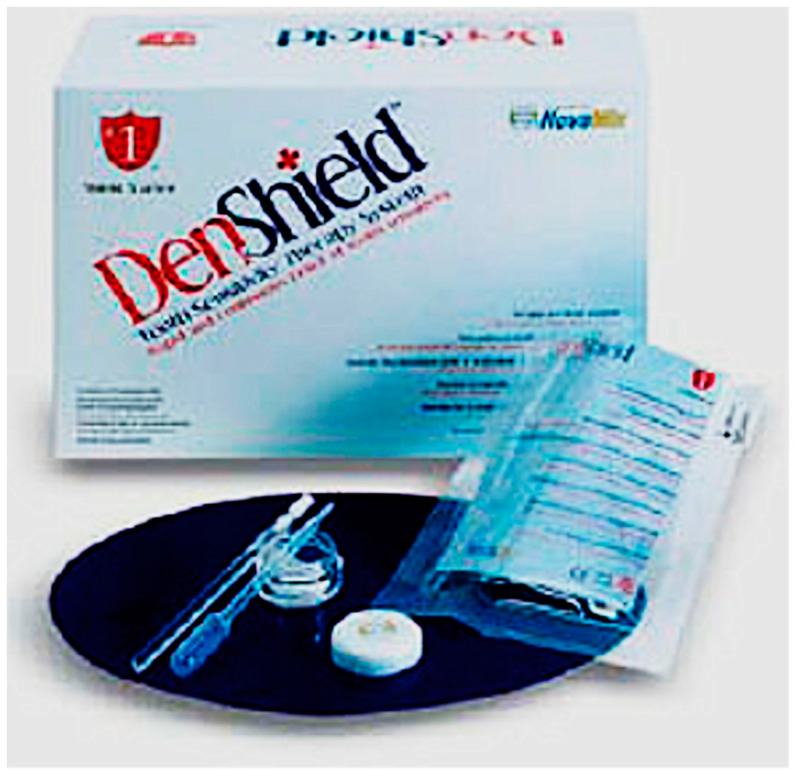
NovaMin^®^ paste prepared by mixing pure novamin powder (Denshield; Shield Starter) and with distilled water.

The NovaMin^®^ paste was prepared by using pure NovaMin^®^ powder (Denshield; NovaMin^®^ Technology, Alachua, FL, USA, Shield Starter) (Figure 1) and mixing it with distilled water to form a mono-structured paste. Pumice paste was prepared by using a fine pumice powder and mixing it with distilled water to give a similar paste.

### 2.4. Assessment of Dentin Hypersensitivity

**Figure 2:**
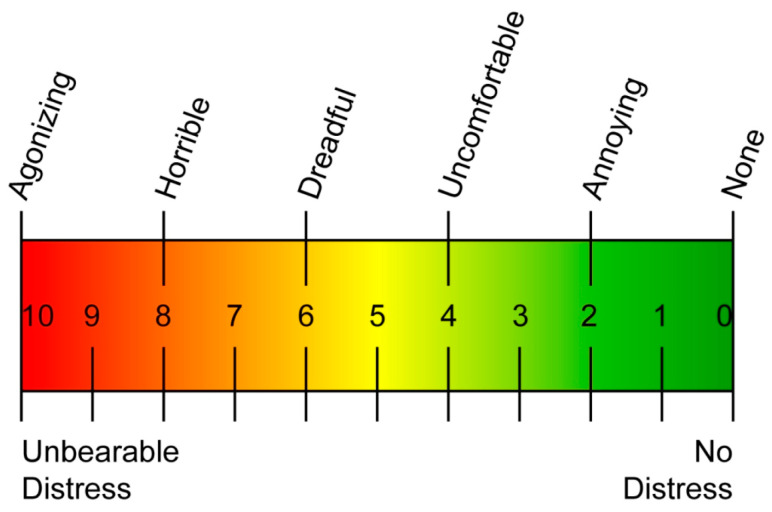
Visual analogue scale (VAS).

**Figure 3:**
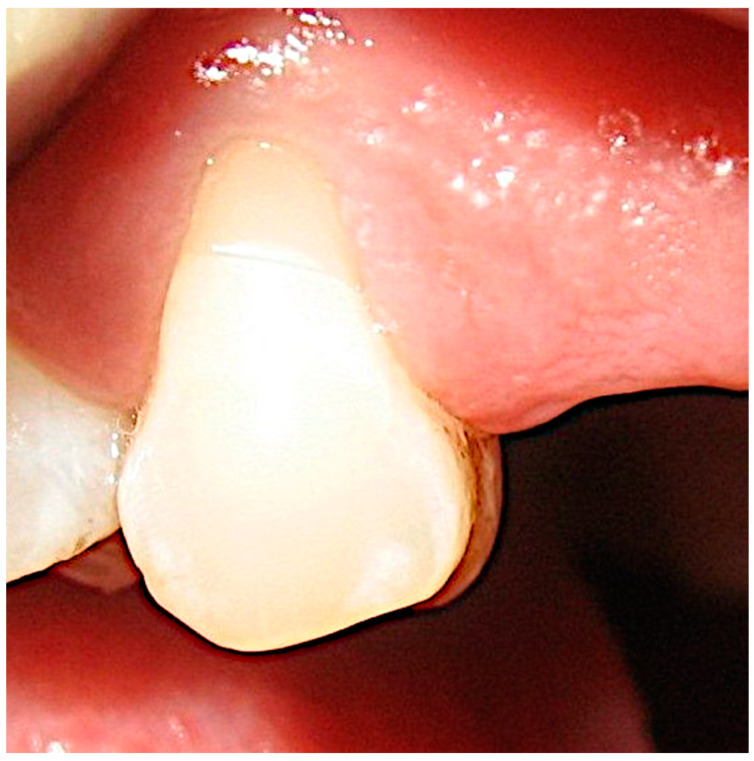
Selection of the most sensitive tooth in the oral cavity.

Sensitivity was measured using a metered air blast of a five-second duration on a visual analogue scale (VAS) with a rating of 0 to 10 [38] (Figure 2). Subjects having at least one tooth sensitive to air stimulation with a VAS rating of 3 or more were included in the study (T1). All 50 subjects included in the study had a VAS rating of 3 or more. The most sensitive tooth in the oral cavity was selected (Figure 3). Exclusion criteria included patients already using desensitizing toothpaste or mouthwashes which may alter the study, severe wasting disease of teeth, restorations in the quadrant of the study and extreme dentinal hypersensitivity which may require restorative or endodontic treatment. Before the start of the treatment, both groups received a thorough ultrasonic scaling. Sensitivity was re-assessed after ultrasonic scaling again (T1).

### 2.5. Study Procedure

A total of fifty subjects were randomly divided into two equal intervention groups using a lottery approach, with Group A subjects receiving NovaMin^®^ as the intervention (*n* = 25) and Group B receiving Pumice (*n* = 25). The intervention groups were only known to one researcher, and the subjects were given identifying codes. For the subsequent follow-up visits, the VAS assessment was carried out by the same researcher.

**Figure 4:**
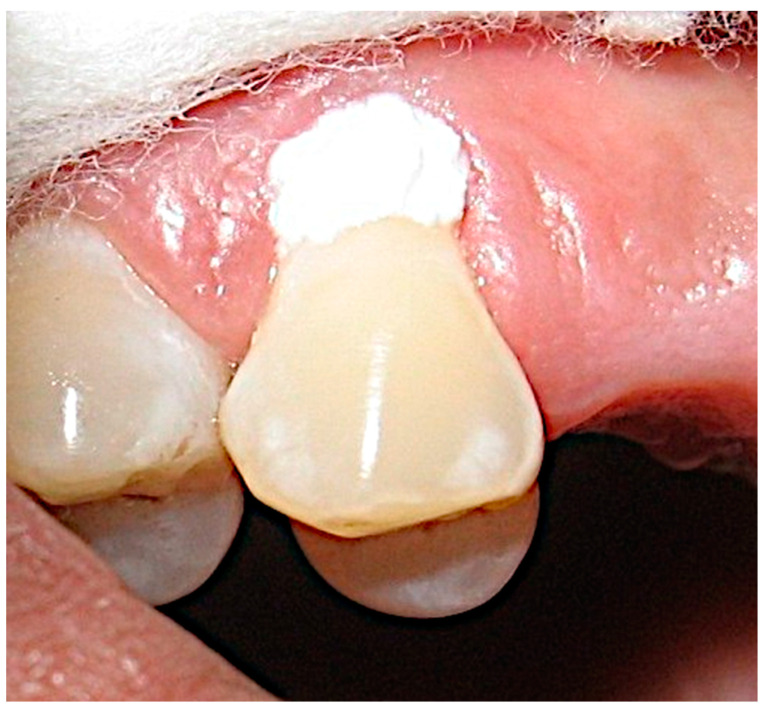
Application of the NovaMin^®^ paste on the most sensitive tooth.

The NovaMin^®^ powder was mixed with distilled water to get a smooth paste-like consistency. The identified tooth in the test group was isolated using cotton rolls and dried using a blast of air for 5 s leaving the tooth moist but not wet. The NovaMin^®^ paste was gently applied on the tooth surface and left for 2 min which allows mineralization to take place (Figure 4). Then the tooth was irrigated lightly with water from a three-way syringe for 15 to 20 s until all the NovaMin^®^ paste was removed. Dentinal hypersensitivity was re-evaluated (T2). Similarly, the control group received a placebo made from mixing pumice and distilled water with a similar consistency as the NovaMin^®^ paste and the prepared paste was applied to the isolated sensitive tooth, left for 2 min, and irrigated lightly to remove the pumice mixture. Sensitivity was re-evaluated again (T2).

The subjects were advised not to eat or drink anything for the next 30 min. Patients were given oral hygiene instructions and recalled after 1, 2 and 4 weeks to reassess the sensitivity (T3, T4 and T5).

Every participant finished the research, and no one experienced any problems at the follow-up sessions. Desensitizing agents were prescribed to patients who felt the need for their use at the completion of the research.

### 2.6. Statistical Analysis

First, the data collected was entered into an MS-Excel spreadsheet, and then was subjected to analysis using SPSS for Windows, Version 16.0. USA, Chicago, Illinois: SPSS Inc. Frequency, mean, and standard deviation were used to show the results in a descriptive manner. The study used Freidman’s test, the Mann—Whitney test, and the Wilcoxon signed-rank post hoc test for statistical analysis in order to compare the mean VAS scores between the two groups, between time intervals, the mean difference between the two groups’ mean VAS scores, and the mean percentage increase between the test and control groups’ mean VAS scores.

## 3. Results

The results show that ultrasonic scaling increases the amount of dentinal sensitivity from an average VAS score of 3.46 to a score of 4.96. The average VAS score in the test group fell from 5.04 to 0.96 immediately following the NovaMin^®^ application and increased slightly to 1.76 one week postoperatively, 2.04 two weeks postoperatively and 1.92 four weeks postoperatively. The control group also showed a slight fall in VAS scores, from an average VAS score of 4.88 to an average VAS score of 3.76 with the follow-up VAS scores being 3.84 one week postoperatively, 3.6 two weeks postoperatively and 3.08 four weeks postoperatively (Table 1).

The percentage of reduction of dentinal hypersensitivity in the test group was about 80.95% immediately, 65.08% one week postoperatively, 59.52% two weeks postoperatively and 61.1% four weeks postoperatively. The percentage of reduction of dentinal hypersensitivity in the control group was about 22.95% immediately, 21.31% one week postoperatively, 26.23% two weeks postoperatively and 36.88% four weeks postoperatively (Table 1).

**Figure 5:**
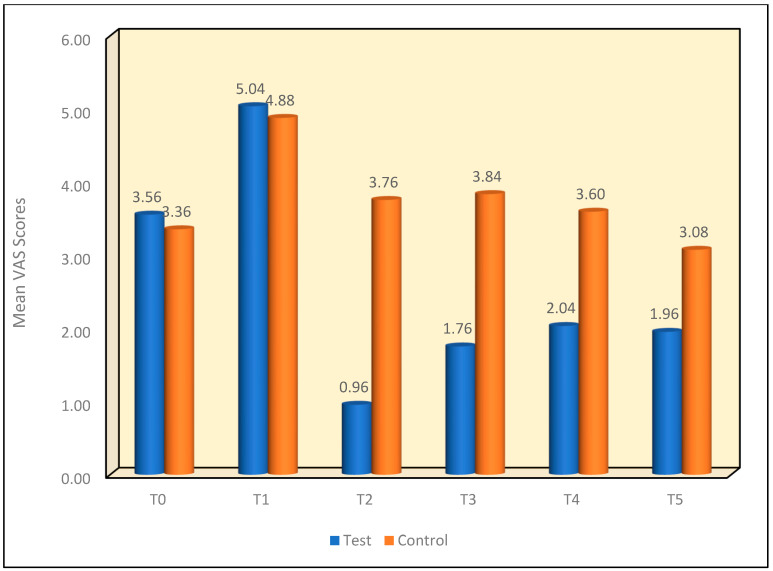
Mean VAS scores between two groups at different time intervals.

The Mann—Whitney test, performed for different time intervals, showed that there was no significant difference in the mean VAS scores between Test and Control group at T0 & T1 time intervals, at *p* = 0.48 & 0.68, respectively. However, at the T2 to T5 time intervals, the test group showed a significant decrease in the mean VAS scores as compared to the control group, and the mean difference in the VAS scores was statistically significant at *p* < 0.001 (Figure 5).

A comparison of the mean VAS scores between various time intervals using Freidman’s test also gave a statistically significant result at *p* < 0.001 between different time frames. (Table 2).

Multiple comparisons of the mean difference in VAS scores in the test group between different time intervals using the Wilcoxon signed-rank post hoc test demonstrated that the mean VAS scores showed a significant increase from the T0 to T1 time interval at *p* < 0.001. Furthermore, there was a significant reduction noted from the T2 to T5 time intervals at *p* < 0.001. This was next followed by the T2 to T5 time intervals showing significantly lower mean VAS scores as compared to T1 at *p* < 0.001. Similarly, the T2 time interval showed significantly lower mean VAS scores as compared to the T3 to T5 time intervals at *p* ≤ 0.001. This was followed with the T3 time interval which showed significantly lower mean VAS scores as compared to the T4 time interval at *p* = 0.008. However, no significant difference was noted between T3 and T5 [*p* = 0.25] or between the T4 and T5 time intervals [*p* = 0.62]. (Table 3).

A comparison of mean VAS scores between time intervals in the control group using Friedman’s test showed that there was a significant difference in the mean VAS scores between different time intervals in the control group at *p* < 0.001. (Table 4).

Multiple comparisons of the mean difference in VAS scores between time intervals in the control group using the Wilcoxon signed-rank post hoc test demonstrated that the mean VAS scores showed a significant increase from the T0 to T1 time interval at *p* < 0.001. Furthermore, a significant reduction was noted from the T2 to T3 time intervals at *p* = 0.04 & *p* = 0.01, respectively. This was next followed by the T2 to T5 time intervals showing significantly lower mean VAS scores as compared to T1 at *p* < 0.001. Similarly, the T2 time interval showed significantly lower mean VAS scores as compared to the T5 time interval at *p* = 0.001. This was followed with the T5 time interval showing significantly lower mean VAS scores as compared to the T3 & T4 time intervals at *p* < 0.001 and *p* = 0.002. However, no significant difference was noted between T0 and the T4 and T5 time intervals [*p* = 0.24 and *p* = 0.11, respectively] or between the T2 and T3 and T4 time intervals [*p* = 0.53 and *p* = 0.29, respectively] and between the T3 & T4 time interval at *p* = 0.06. (Table 5).

**Figure 6:**
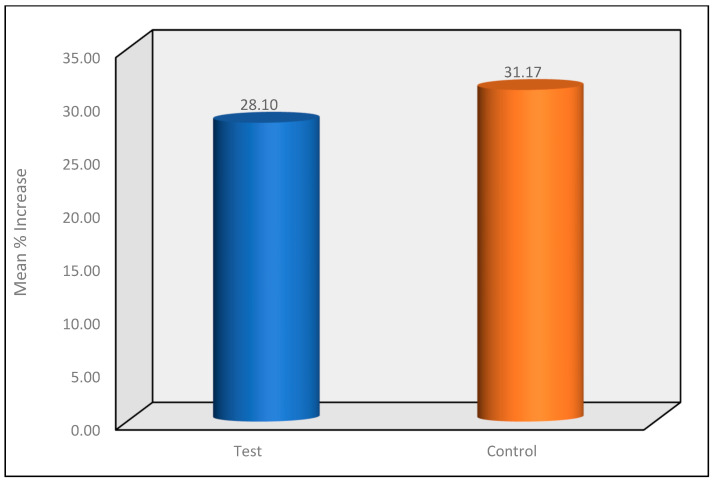
Mean percentage increase in VAS between the two groups at the T1 Time Interval.

A comparison of the mean percentage increase in VAS between the two groups at the T1 Time Interval was carried out using Mann—Whitney Test. The mean percentage increase in the VAS scores from the T0 to T1 time interval showed that the test group showed a relatively lower mean increase in VAS scores as compared to the control group. However, the difference in the mean percentage increase in VAS scores between the two groups was not statistically significant at *p* = 0.53. (Figure 6).

**Figure 7:**
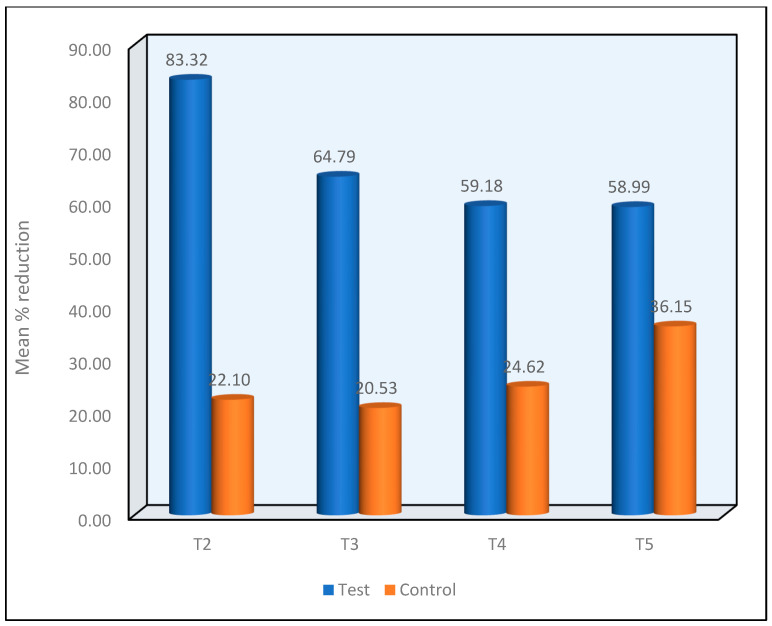
Mean percentage reduction in VAS between the two groups at the T2 to T5 Time Intervals.

A comparison of the mean percentage decrease in VAS between the two groups at the T2 to T5 Time Intervals using the Mann—Whitney Test revealed that the test group demonstrated a significantly higher mean percentage reduction in VAS scores as compared to the control group and the difference between the two groups at the T2 to T5 time intervals was statistically significant at *p* < 0.001. (Figure 7).

The results show that a single chair-side application of NovaMin^®^ in powder form significantly reduced dentinal hypersensitivity immediately after application and the effect lasted until the 4-week post-operative follow-up.

## 4. Discussion

Dentin sensitivity is a chronic condition and may be one of the most common painful conditions of the oral cavity; yet it is one of the least satisfactorily treated. The prevalence of dentin sensitivity is likely to increase as the adult population lives longer and retains their teeth later in life, and as populations of all age groups engage in lifestyles and behaviors that promote dentin exposure through gingival recession or erosion of protective tooth surfaces. A survey among oral care professionals reports that dental care providers feel confident about diagnosing dentin hypersensitivity but not about treating it [2]. Studies show that the incidence of dentinal sensitivity increases after supra or sub-gingival scaling. Different theories have been proposed to explain the concept of dentin hypersensitivity. The Brannstrom theory which deals with the flow of fluid inside the dentinal tubules is currently accepted as a valid theory [11,12]. Dentine sensitivity is generally measured using visual analogue scales (VAS) in many clinical situations. VAS have proved to be a good tool for the measurement of dentinal sensitivity as pain is subjective and can vary greatly among different individuals.

Clinicians dealing with dentin hypersensitivity would benefit greatly from the development of a therapy that offers both rapid relief following expert administration and a long-lasting desensitizing impact. Increased patient compliance with professional oral care recommendations and greater acceptance of advice to address their additional oral care needs are both influenced by a clinician’s ability to give improved oral comfort.

The initial purpose of the highly water-reactive NovaMin^®^ substance was to regenerate bone. Because it releases the calcium and phosphate ions necessary for the synthesis of biologically active hydroxycarbonate apatite, which is like the mineral component of teeth and bones, it is beneficial in the treatment of hard tissues. For more than 15 years, multiple bone generation/replacement surgical treatments have effectively utilized the same compound, and processed into diverse physical forms [39,40].

Initial in vitro research clearly showed that the ions released from NovaMin^®^, when used as a dentifrice [41] or in powder form [42,43], would form hydroxycarbonate apatite, which resembled or was identical to a normal tooth mineral formed by salivary ions. This new layer, along with the particles themselves, effectively occluded exposed dentin tubules [44,45]. Arginine and calcium are positively charged substances that bind to dentin surfaces at physiological pH to create a calcium-rich coating that blocks and seals exposed tubules. According to a study, desensitizing dentifrices with 15% n-hydroxy apatite can effectively obstruct tubules, reducing DH-related pain and discomfort [46,47].

There are few in vivo studies demonstrating the success of NovaMin^®^ in dentifrice form. The results demonstrated rapid and continuous relief from tooth hypersensitivity when used over a period [10,11,20,21].

In this study, a single chair-side application of NovaMin^®^ in powder form was evaluated as a treatment modality for immediate relief of isolated cervical dentinal hypersensitivity based on VAS scores over a period of four weeks.

The average VAS scores for the entire group were 3.46 before the start of the study which changed to 4.96 after the completion of ultrasonic scaling: an immediate increase in sensitivity after scaling. This is also supported by a few other authors who got similar results in their studies [13,14,15,16,17].

The VAS score in the test group dropped from 5.04 to 0.96 after the application of NovaMin^®^ paste demonstrating the efficacy of NovaMin^®^ in reducing dentinal sensitivity. The scores increased slightly in the follow-up visits i.e., 1.76, 2.04 and 1.96 but remained below the initial scores. There was no significant difference seen in the control group although the scores dropped a bit.

Reduction of dentinal sensitivity in the test group may be attributed to the penetration of the NovaMin^®^ molecules into the dentinal tubule, reacting with the salivary ions and resulting in the precipitation of calcium and phosphate to form a hard layer of calcium phosphate on the tooth surface which later crystalizes forming the hydroxycarbonate apatite crystal resulting in a barrier between the tooth and the oral environment, thus occluding the tubules and reducing dentinal sensitivity [28,29]. This study also supports the previous in vitro studies that have demonstrated superior tubular occlusion as evident on the Scanning Electron Microscope (SEM) [42]. The results of the present study were in support of previous in vitro/in vivo studies [28,29,30,31,32,33,34,35,36,37,41,44,45,46,47,48].

However, the use of NovaMin^®^ in powder form for the treatment of dentinal hypersensitivity must be further evaluated with a large sample size for long-term benefits.

## 5. Conclusions

The use of NovaMin^®^ in paste form as described in this study can be a good chair-side technique for rapid relief from dentinal hypersensitivity. Further studies with a bigger sample size over an extended period of time may be required to support the efficacy of NovaMin^®^ as a novel single-use technique for rapid relief from dentinal sensitivity.

## Figures and Tables

**Table 1:** VAS reading at different time intervals in two groups. (T0–Before Scaling; T1–After Scaling; T2–Post Procedure, T3–Post 1 Week, T4–Post 2 weeks and T5–Post 4 weeks).

Test Group							Control Group					
Time Interval	T0	T1	T2	T3	T4	T5	T0	T1	T2	T3	T4	T5
Patient-1	2	3	0	1	1	1	2	4	3	3	3	2
Patient-2	2	4	2	3	3	2	2	4	3	2	2	2
Patient-3	2	4	1	1	2	2	5	6	4	5	5	4
Patient-4	4	6	2	3	3	4	3	4	3	3	3	3
Patient-5	4	5	1	2	2	3	2	5	3	3	2	2
Patient-6	4	4	0	1	2	2	2	3	2	2	3	3
Patient-7	2	3	0	1	1	2	4	6	5	4	4	4
Patient-8	4	4	0	1	1	2	6	7	5	5	4	5
Patient-9	5	6	2	3	3	2	5	5	5	5	5	4
Patient-10	3	5	1	2	2	2	2	4	4	4	4	3
Patient-11	3	7	3	2	2	1	4	5	3	4	4	4
Patient-12	3	4	0	1	1	2	5	6	4	4	4	3
Patient-13	5	7	2	2	3	2	5	7	5	5	4	4
Patient-14	4	7	1	2	2	2	3	3	2	3	2	1
Patient-15	4	4	0	2	2	2	4	5	5	5	4	3
Patient-16	4	5	1	2	2	2	2	3	3	3	3	3
Patient-17	4	6	0	3	3	2	2	4	3	3	3	2
Patient-18	3	6	2	1	2	2	3	3	3	2	2	2
Patient-19	5	7	1	2	2	1	4	7	5	5	4	3
Patient-20	2	4	0	1	1	1	3	6	4	5	5	4
Patient-21	3	4	0	1	2	1	3	5	5	4	5	3
Patient-22	4	5	1	2	3	2	2	3	2	3	3	2
Patient-23	5	6	2	3	3	2	2	4	3	3	2	2
Patient-24	5	7	2	1	2	3	5	7	5	6	5	5
Patient-25	3	3	0	1	1	2	4	6	5	5	5	4
Mean	3.56	5.04	0.96	1.76	2.04	1.96	3.36	4.88	3.76	3.84	3.6	3.08
SD	1.04	1.367	0.935	0.78	0.73	0.68	1.29	1.39	1.09	1.143	1.080	1.039
% Change	41.57	0	80.95	65.08	59.52	61.11	31.15	0	22.95	21.311	26.23	36.88

**Table 2:** Comparison of mean VAS scores between time intervals in the test group using Friedman’s test.

Time	*N*	Mean	SD	Min	Max	*p*-Value
T0	25	3.56	1.04	2	5	<0.001 *
T1	25	5.04	1.37	3	7	
T2	25	0.96	0.94	0	3	
T3	25	1.76	0.78	1	3	
T4	25	2.04	0.74	1	3	
T5	25	1.96	0.68	1	4	

*—Statistically significant.

**Table 3:** Multiple comparisons of mean difference in VAS scores b/w time intervals in test group using Wilcoxon signed-rank post hoc test.

(I) Time	(J) Time	Mean Diff. (I − J)	95% CI for the Diff.		*p*-Value
			Lower	Upper	
T0	T1	−1.48	−2.14	−0.83	<0.001 *
	T2	2.60	1.85	3.35	<0.001 *
	T3	1.80	1.15	2.45	<0.001 *
	T4	1.52	0.92	2.12	<0.001 *
	T5	1.60	0.92	2.28	<0.001 *
T1	T2	4.08	3.46	4.70	<0.001 *
	T3	3.28	2.49	4.07	<0.001 *
	T4	3.00	2.27	3.73	<0.001 *
	T5	3.08	2.16	4.00	<0.001 *
T2	T3	−0.80	−1.36	−0.24	<0.001 *
	T4	−1.08	−1.58	−0.59	<0.001 *
	T5	−1.00	−1.65	−0.35	0.001 *
T3	T4	−0.28	−0.58	0.02	0.008 *
	T5	−0.20	−0.76	0.36	0.25
T4	T5	0.08	−0.45	0.61	0.62

*—Statistically significant.

**Table 4:** Comparison of mean VAS scores between time intervals in the control group using Friedman’s test.

Time	*N*	Mean	SD	Min	Max	*p*-Value
T0	25	3.36	1.29	2	6	<0.001 *
T1	25	4.88	1.39	3	7	
T2	25	3.76	1.09	2	5	
T3	25	3.84	1.14	2	6	
T4	25	3.60	1.08	2	5	
T5	25	3.08	1.04	1	5	

*—Statistically significant.

**Table 5:** Multiple comparison of mean difference in VAS scores b/w time intervals in the control group using the Wilcoxon signed-rank post hoc test.

(I) Time	(J) Time	Mean Diff. (I − J)	95% CI for the Diff.		*p*-Value
			Lower	Upper	
T0	T1	−1.52	−2.09	−0.95	<0.001 *
	T2	−0.40	−1.00	0.20	0.04 *
	T3	−0.48	−1.02	0.06	0.01 *
	T4	−0.24	−0.90	0.42	0.24
	T5	0.28	−0.27	0.83	0.11
T1	T2	1.12	0.61	1.63	<0.001 *
	T3	1.04	0.56	1.52	<0.001 *
	T4	1.28	0.61	1.95	<0.001 *
	T5	1.80	1.21	2.40	<0.001 *
T2	T3	−0.08	−0.50	0.34	0.53
	T4	0.16	−0.33	0.65	0.29
	T5	0.68	0.16	1.20	0.001 *
T3	T4	0.24	−0.15	0.63	0.06
	T5	0.76	0.29	1.23	<0.001 *
T4	T5	0.52	0.09	0.95	0.002 *

*—Statistically significant.

## Data Availability

Data can be made available on demand by the chief researcher for academic purposes by email.
